# Supplementary figures and images for: New taxonomic insights for Brazilian Syrbatus Reitter (Coleoptera: Staphylinidae: Pselaphinae), including three new species and their mitochondrial genomes
Source: PeerJ. 2024 Aug 12;12:e17783. doi: 10.7717/peerj.17783 (PMC11326439; doi:10.7717/peerj.17783)

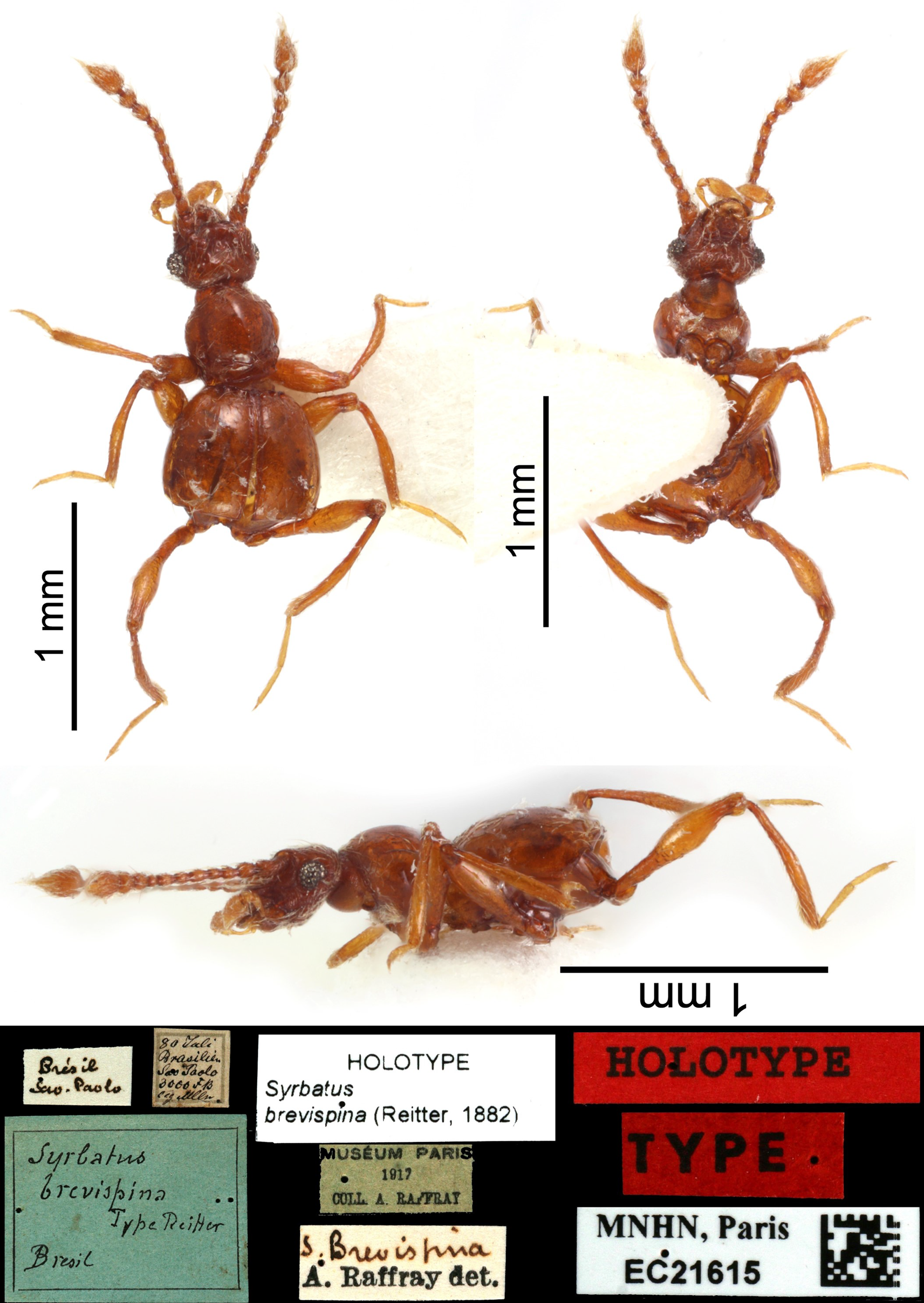

Supplement: Figure S1 — Habitus, dorsal view (A); habitus, ventral view (B); habitus, lateral view (C); labels (D). Photo credit: MNHN/Maéva Pronesti. [file peerj-12-17783-s001.png]

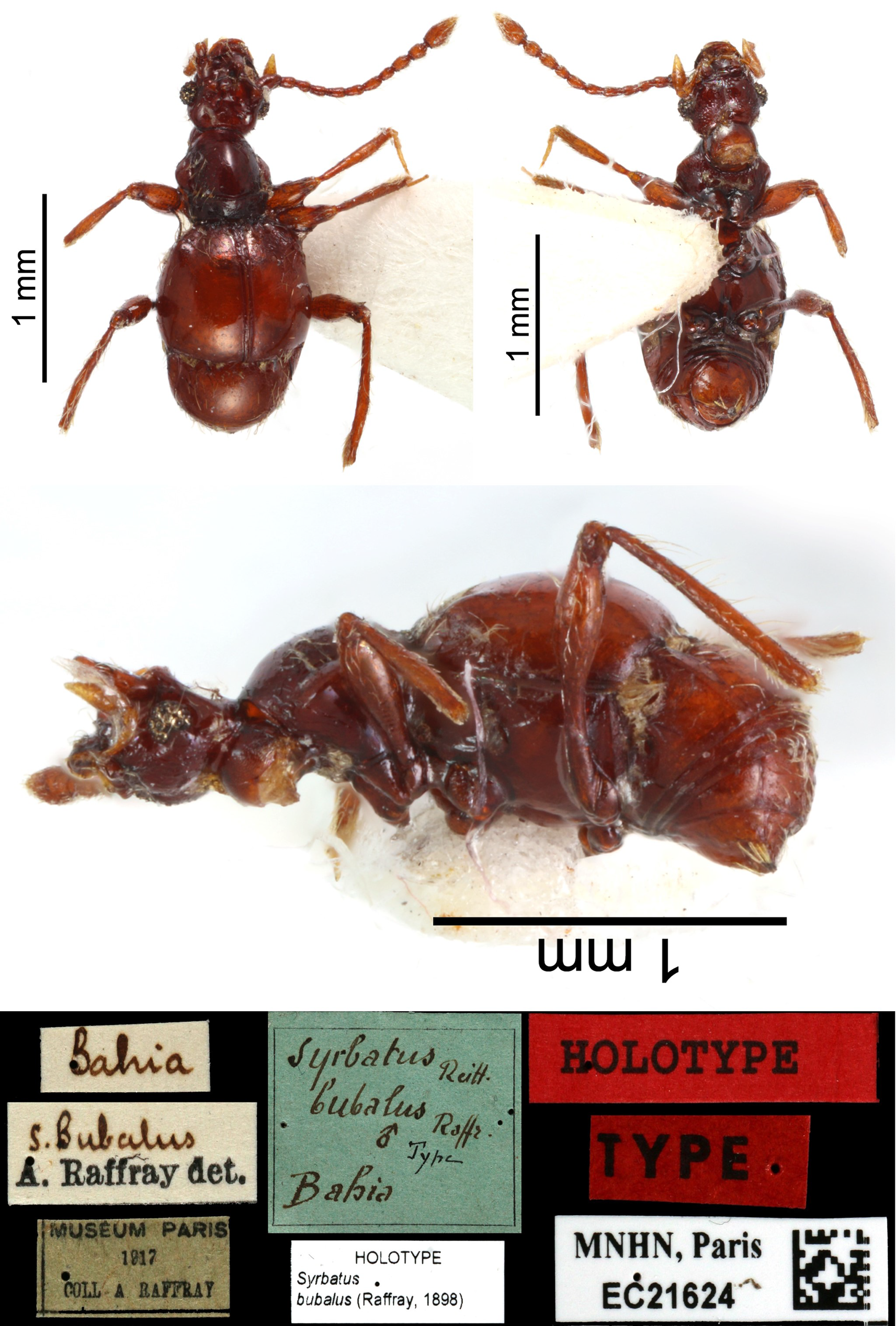

Supplement: Figure S2 — Habitus, dorsal view (A); habitus, ventral view (B); habitus, lateral view (C); labels (D). Photo credit: MNHN/Maéva Pronesti. [file peerj-12-17783-s002.png]

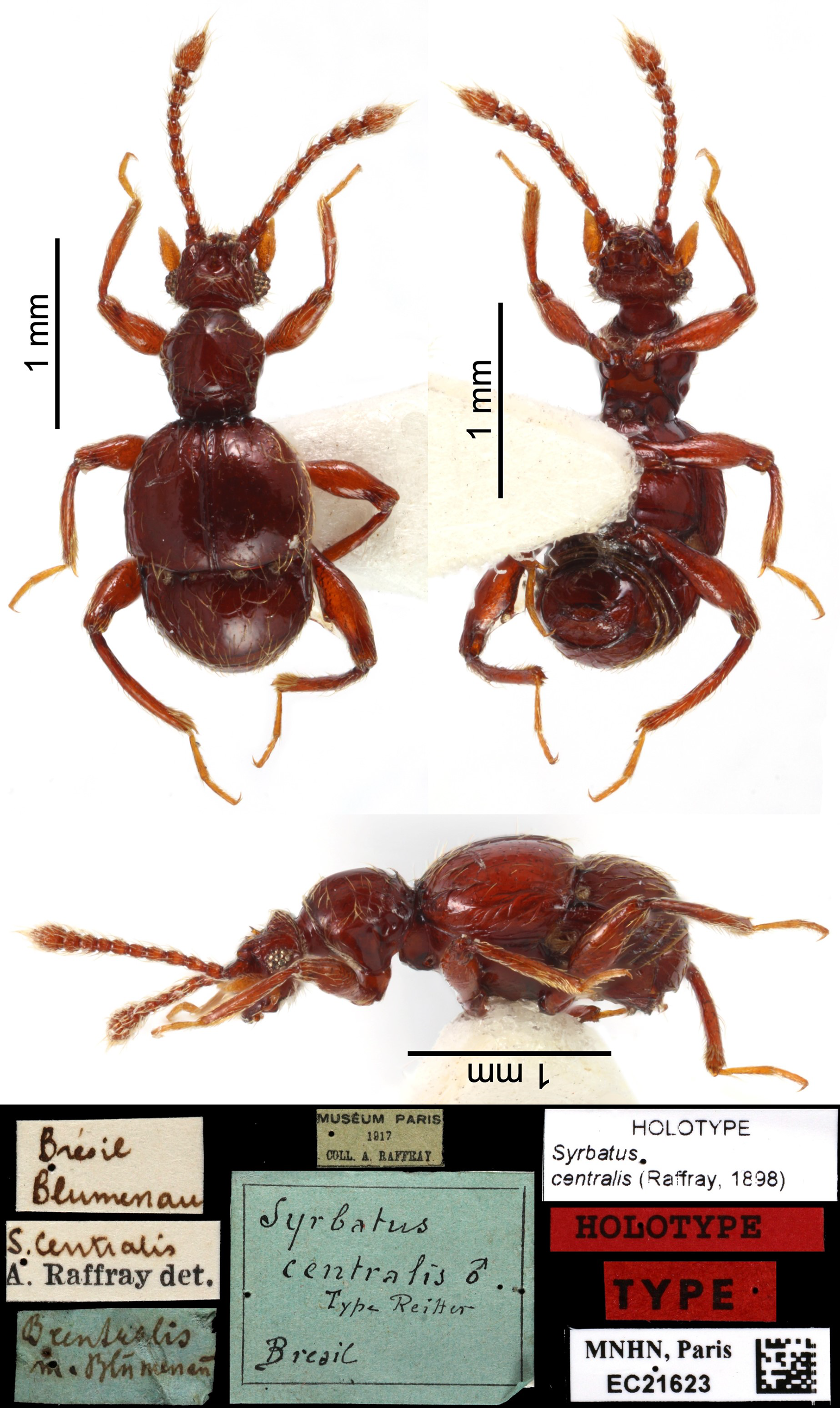

Supplement: Figure S3 — Habitus, dorsal view (A); habitus, ventral view (B); habitus, lateral view (C); labels (D). Photo credit: MNHN/Maéva Pronesti. [file peerj-12-17783-s003.png]

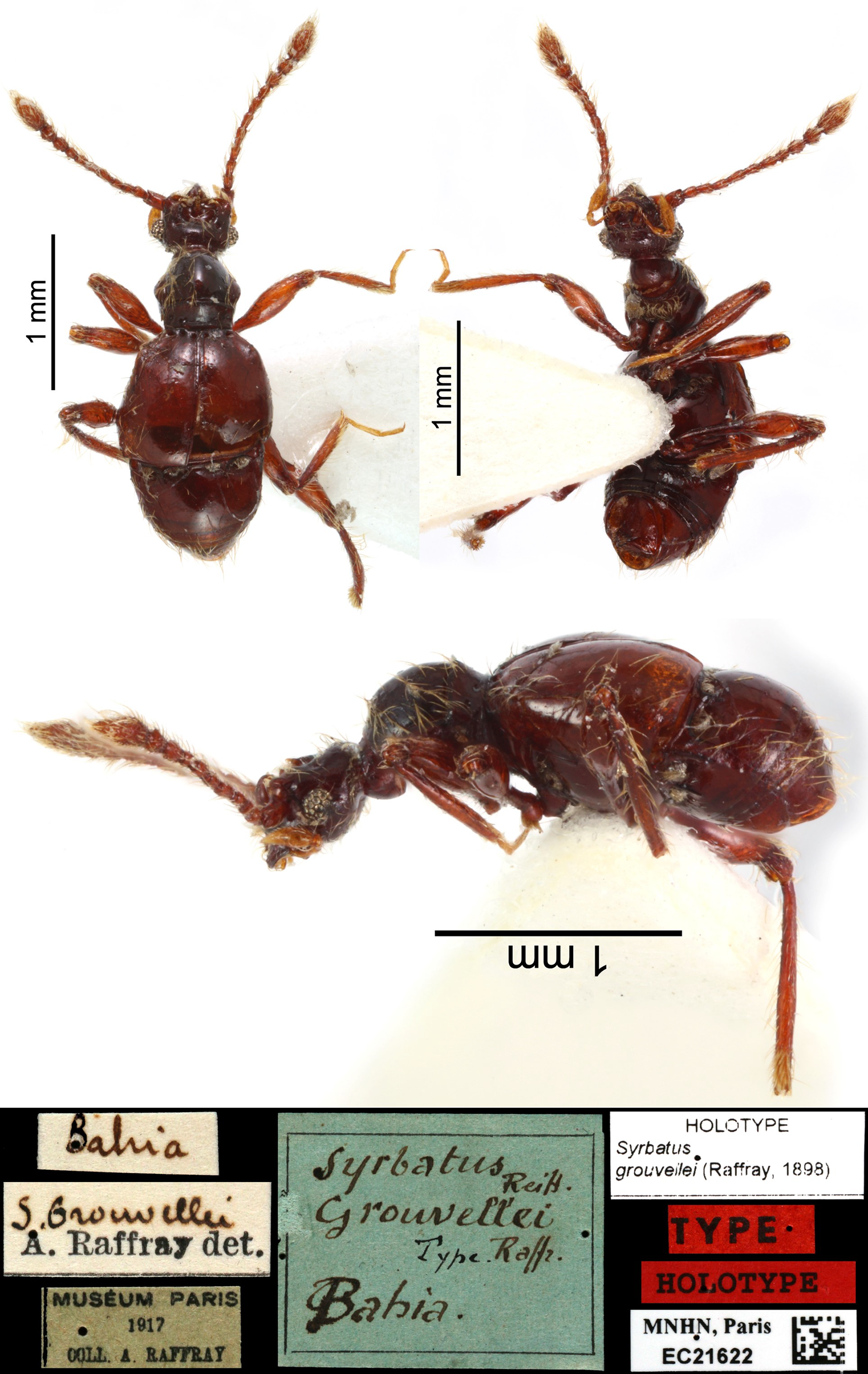

Supplement: Figure S4 — Habitus, dorsal view (A); habitus, ventral view (B); habitus, lateral view (C); labels (D). Photo credit: MNHN/Maéva Pronesti. [file peerj-12-17783-s004.png]

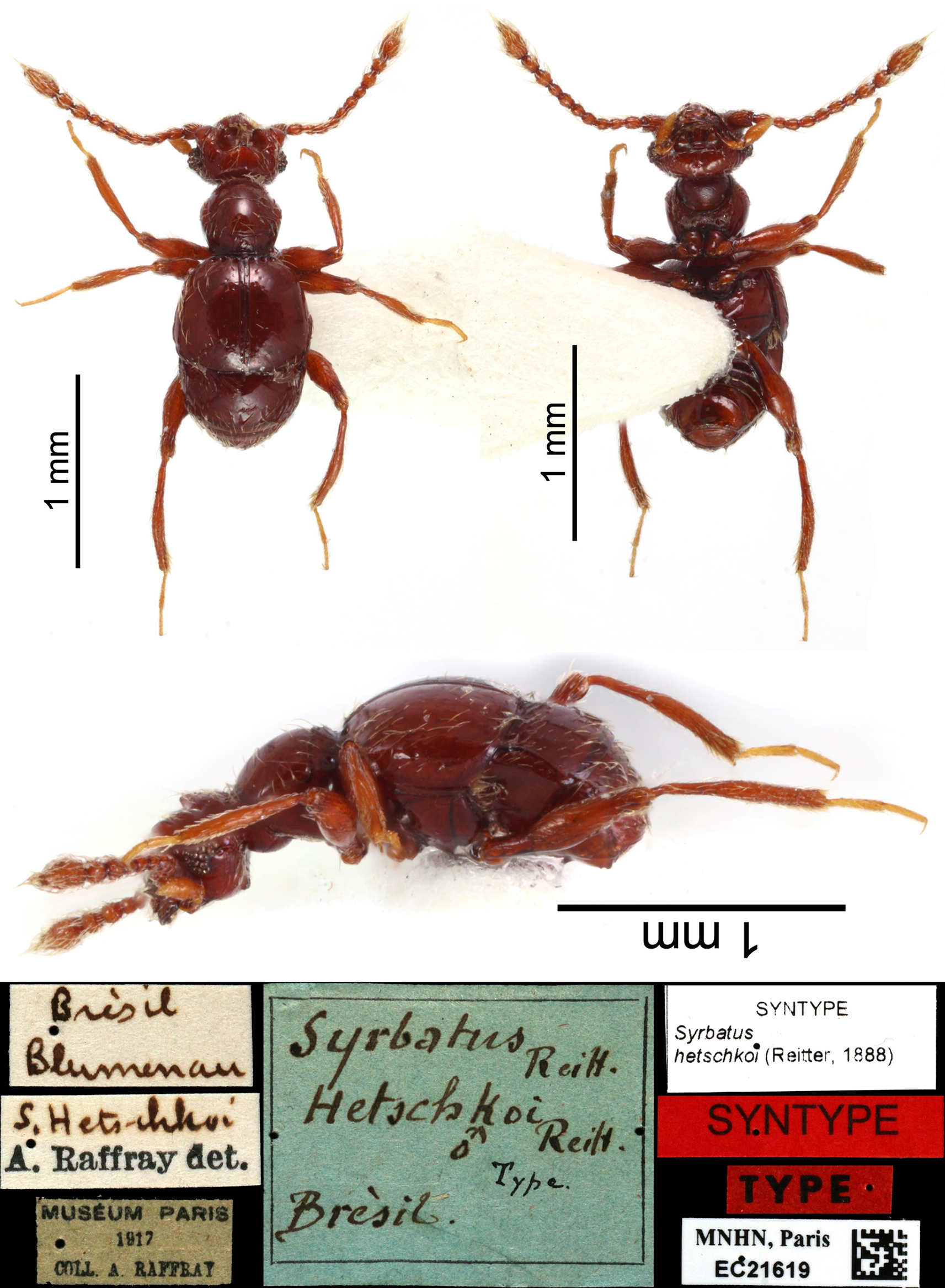

Supplement: Figure S5 — Habitus, dorsal view (A); habitus, ventral view (B); habitus, lateral view (C); labels (D). Photo credit: MNHN/Maéva Pronesti. [file peerj-12-17783-s005.png]

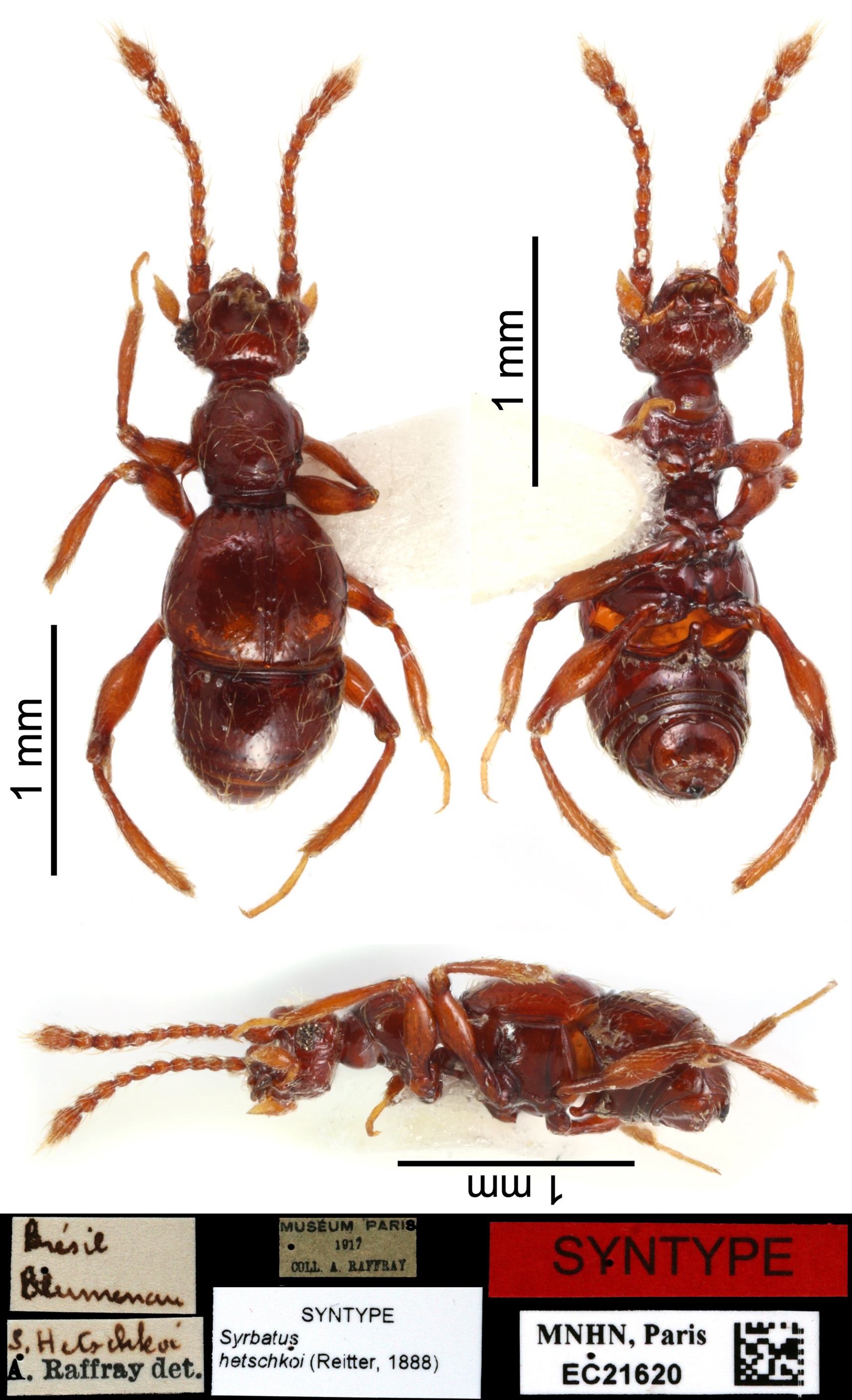

Supplement: Figure S6 — Habitus, dorsal view (A); habitus, ventral view (B); habitus, lateral view (C); labels (D). Photo credit: MNHN/Maéva Pronesti. [file peerj-12-17783-s006.png]

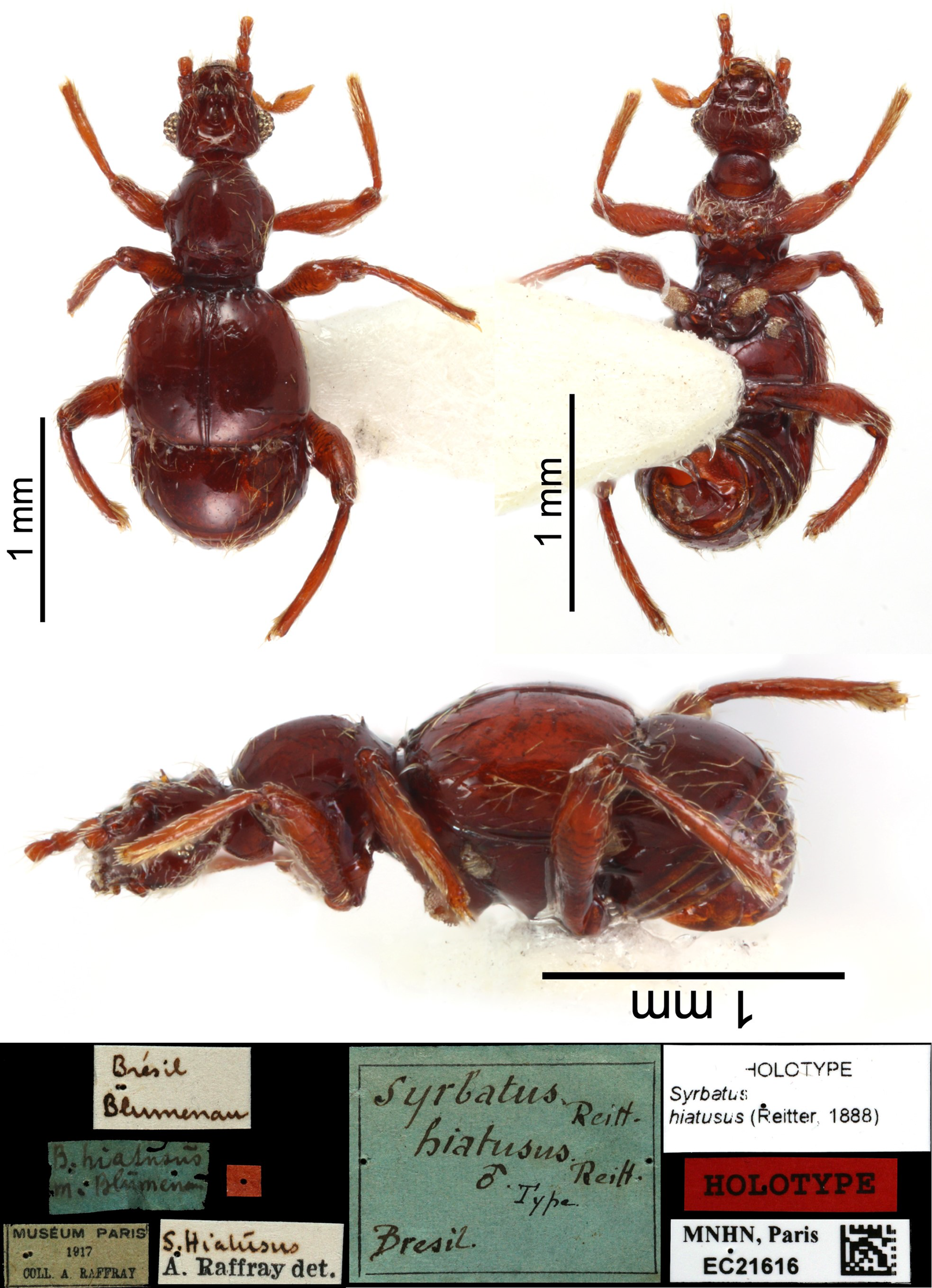

Supplement: Figure S7 — Habitus, dorsal view (A); habitus, ventral view (B); habitus, lateral view (C); labels (D). Photo credit: MNHN/Maéva Pronesti. [file peerj-12-17783-s007.png]

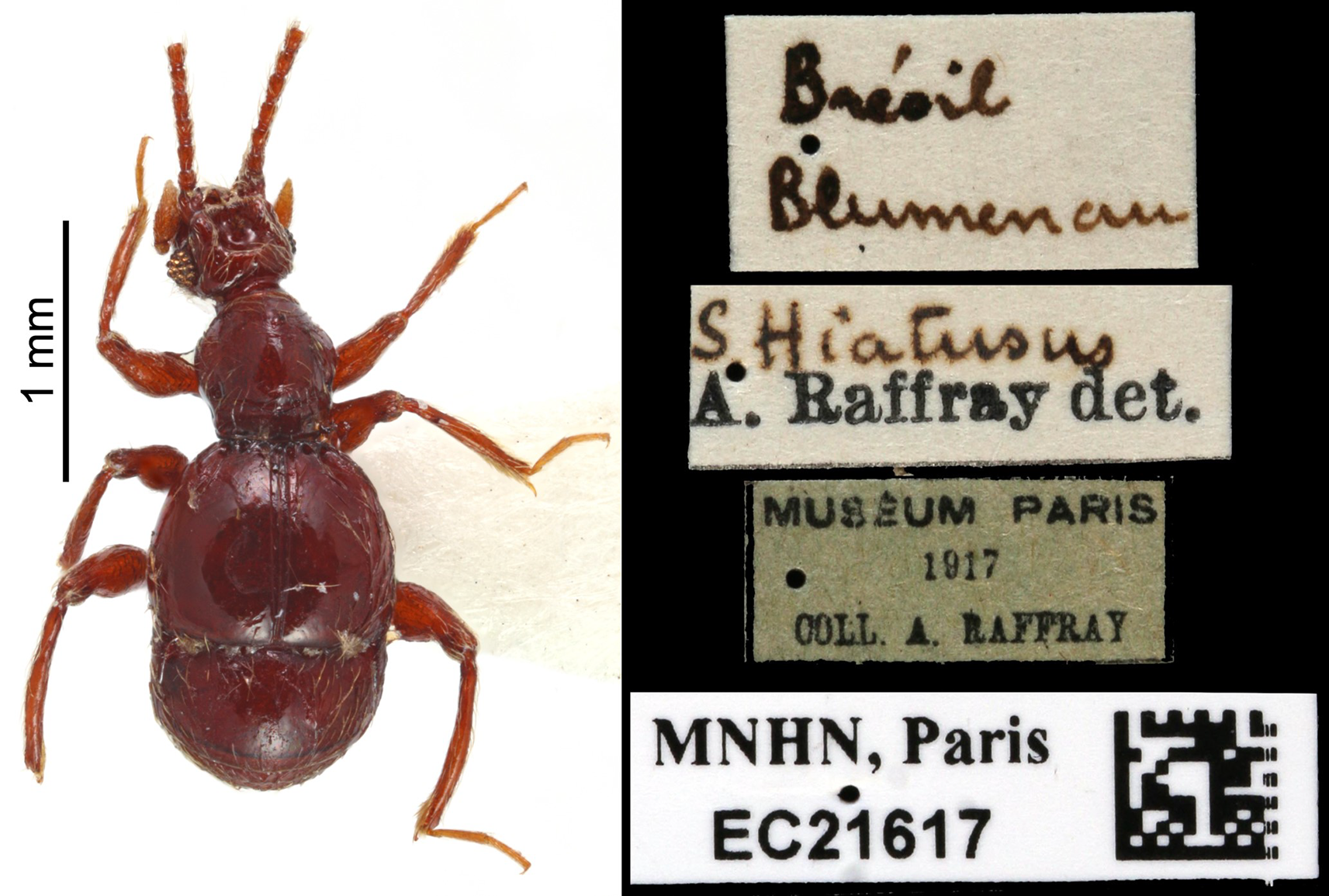

Supplement: Figure S8 — Habitus, dorsal view (A); habitus, ventral view (B); habitus, lateral view (C); labels (D). Photo credit: MNHN/Maéva Pronesti. [file peerj-12-17783-s008.png]

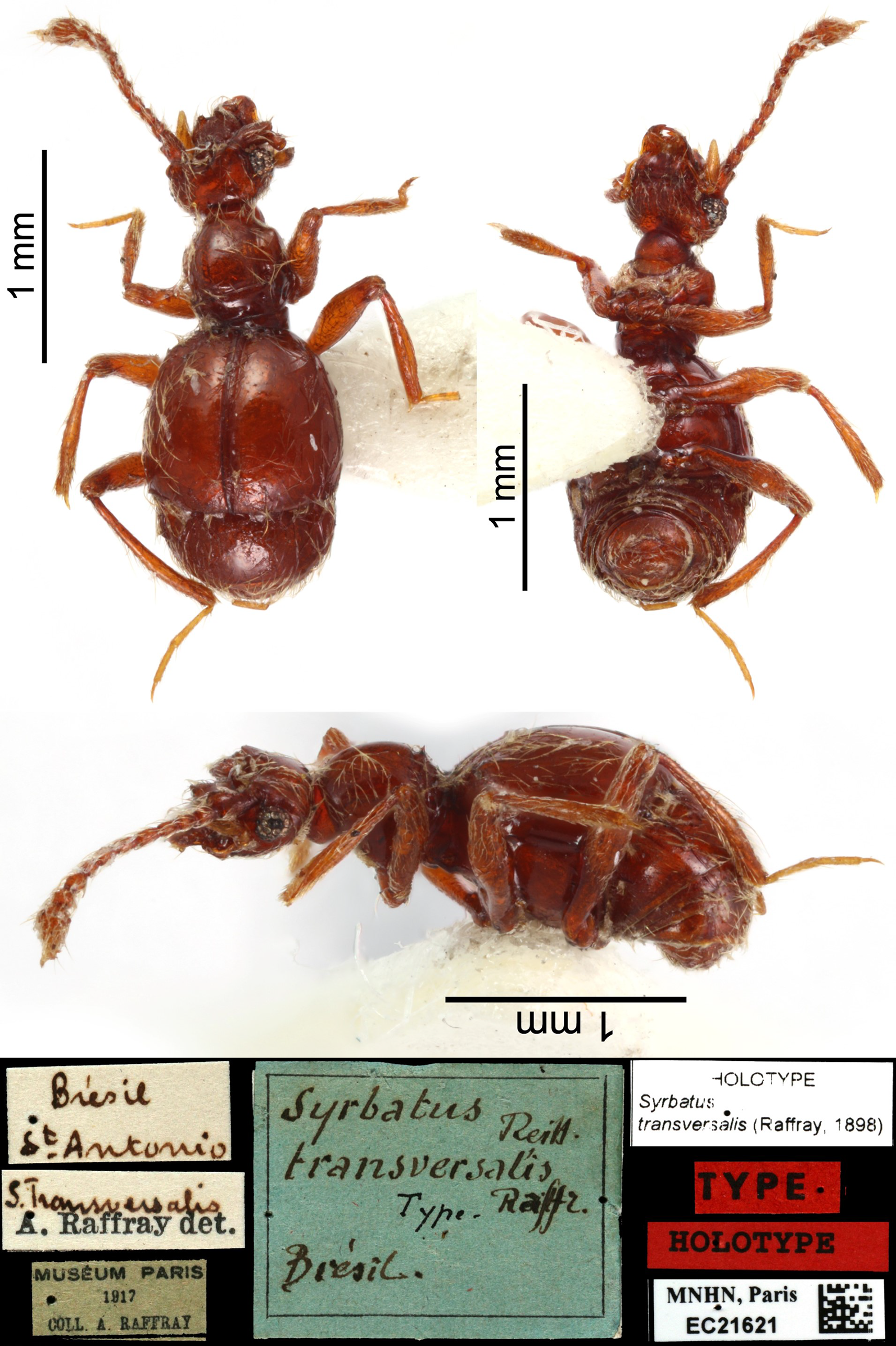

Supplement: Figure S9 — Habitus, dorsal view (A); habitus, ventral view (B); habitus, lateral view (C); labels (D). Photo credit: MNHN/Maéva Pronesti. [file peerj-12-17783-s009.png]

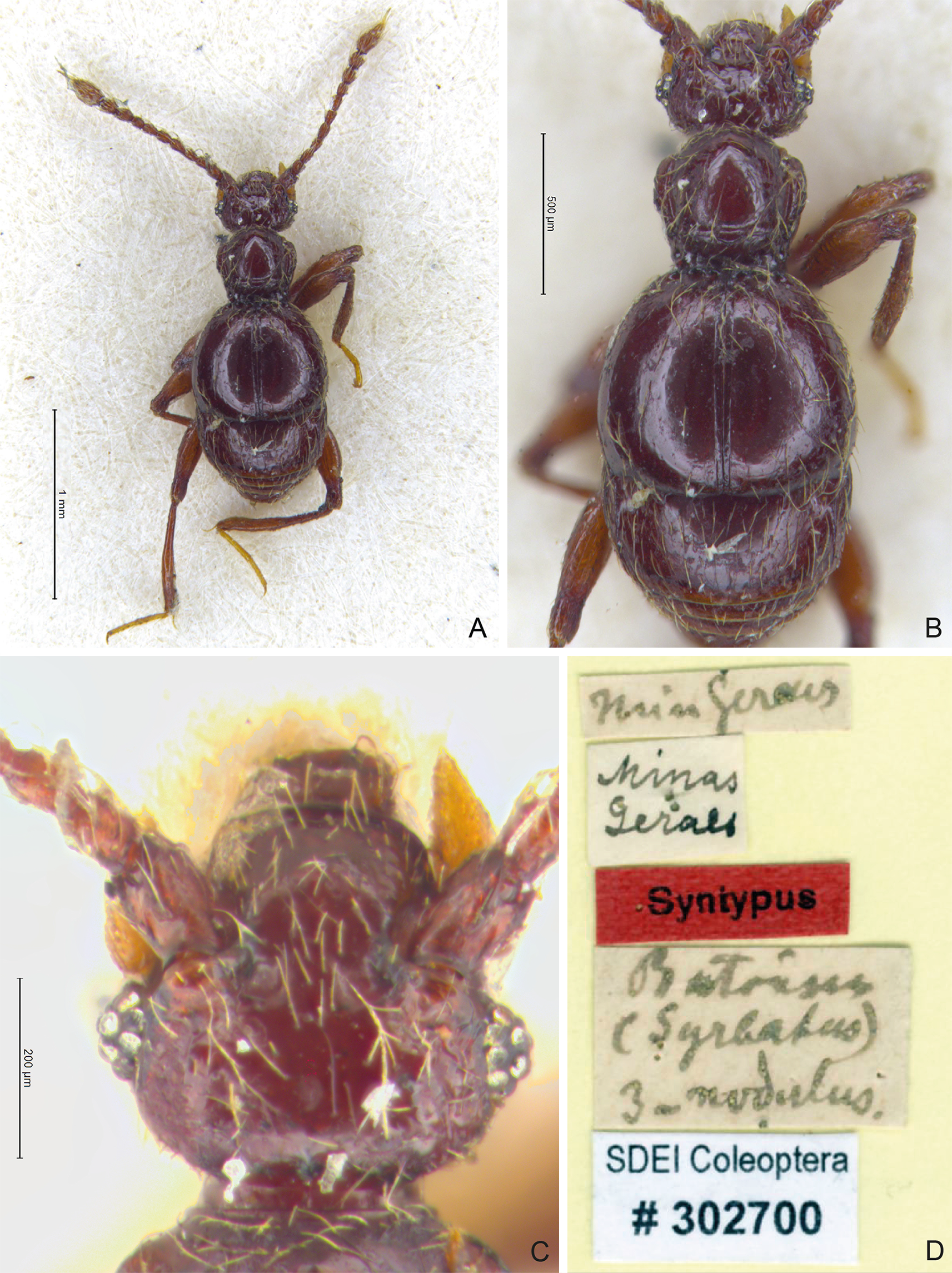

Supplement: Figure S10 — Habitus, dorsal view (A); habitus, ventral view (B); habitus, lateral view (C); labels (D). Photo credit: SDEI/Mandy Schröter. [file peerj-12-17783-s010.png]
