# Supplementary material for: New taxonomic insights for Brazilian Syrbatus Reitter (Coleoptera: Staphylinidae: Pselaphinae), including three new species and their mitochondrial genomes
Source: PeerJ. 2024 Aug 12;12:e17783. doi: 10.7717/peerj.17783 (PMC11326439; doi:10.7717/peerj.17783)
Supplement: Table S1 [file peerj-12-17783-s011.docx]

**Table S1:** **ASAP scores and probability values of the species delimitation analysis with the three new species of *Syrbatus* and *Metopiellus crypticus*.**

| Overall rank | N. of subsets | asap-score | P-val | W | Treshold distance |
| --- | --- | --- | --- | --- | --- |
| 1 | 4 | 1.00 | 0.0019 | 0.0387 | 0.0467 |
| 2 | 5 | 2.50 | 0.0163 | 0.0142 | 0.0159 |
| 3 | 4 | 2.50 | 0.0798 | 0.0175 | 0.1984 |
| 4 | 6 | 4.00 | 0.4430 | 0.0043 | 0.0062 |
| 5 | 7 | 6.00 | 1.0000 | 0.0033 | 0.0011 |
| 6 | 8 | 6.50 | 0.9200 | 0.0002 | 0.0004 |
| 7 | 10 | 7.50 | 1.0000 | 0.0004 | 0.0000 |
| 8 | 9 | 7.50 | 1.0000 | 0.0002 | 0.0002 |
